# Characterization of *Highper*, an ENU-induced mouse mutant with abnormal psychostimulant and stress responses

**DOI:** 10.1007/s00213-012-2827-5

**Published:** 2012-09-05

**Authors:** Amy F. Eisener-Dorman, Janice S. Bailey, Laura Grabowski-Boase, Salvador Huitron-Resendiz, Amanda J. Roberts, Tim Wiltshire, Lisa M. Tarantino

**Affiliations:** 1 Department of Psychiatry, University of North Carolina, Chapel Hill, NC 27599 USA; 2 Present Address: Department of Biological Sciences, Miracosta College, Oceanside, CA USA; 3 Genomics Institute of the Novartis Research Foundation, San Diego, CA USA; 4 Molecular and Integrative Neurosciences Department, The Scripps Research Institute, La Jolla, CA USA; 5 Institute of Pharmacogenomics and Individualized Therapy, Division of Pharmacotherapy and Experimental Therapeutics, University of North Carolina, Chapel Hill, NC USA

**Keywords:** ENU, Mutagenesis, Behavior, Cocaine, Activity, CPP, Self-administration, Alcohol, Methylphenidate, HPA

## Abstract

**Rationale:**

Chemical mutagenesis in the mouse is a forward genetics approach that introduces random mutations into the genome, thereby providing an opportunity to annotate gene function and characterize phenotypes that have not been previously linked to a given gene.

**Objectives:**

We report on the behavioral characterization of *Highper*, an *N*-ethyl-*N*-nitrosourea (ENU)-induced mutant mouse line.

**Methods:**

*Highper* and B6 control mice were assessed for locomotor activity in the open field and home cage environments. Basal and acute restraint stress-induced corticosterone levels were measured. Mice were tested for locomotor response to cocaine (5, 20, 30, and 45 mg/kg), methylphenidate (30 mg/kg), and ethanol (0.75, 1.25, and 1.75 g/kg). The rewarding and reinforcing effects of cocaine were assessed using conditioned place preference and self-administration paradigms.

**Results:**

*Highper* mice are hyperactive during behavioral tests but show normal home cage locomotor behavior. *Highper* mice also exhibit a twofold increase in locomotor response to cocaine, methylphenidate, and ethanol and prolonged activation of the hypothalamic–pituitary–adrenal axis in response to acute stress. *Highper* mice are more sensitive to the rewarding and reinforcing effects of cocaine, although place preference in *Highper* mice appears to be significantly influenced by the environment in which the drug is administered.

**Conclusions:**

Altogether, our findings indicate that *Highper* mice may provide important insights into the genetic, molecular, and biological mechanisms underlying stress and the drug reward pathway.

**Electronic supplementary material:**

The online version of this article (doi:10.1007/s00213-012-2827-5) contains supplementary material, which is available to authorized users.

## Introduction

Addiction is a significant public health concern with serious personal, economic, and societal impacts. Family and twin studies indicate that susceptibility to drug addiction is heritable (Bierut et al. 1998), and a genetic basis for drug abuse and drug response has been established in studies of human populations (Uhl et al. 1995) and in animal models (Crabbe et al. 1994; George and Goldberg 1989; Seale and Carney 1991). Consequently, the identification of genes that contribute to drug abuse susceptibility has become a priority of psychopharmacological research.

Due to the complex etiology of addiction and the inherent difficulties of conducting such studies in human populations, the genetic factors predisposing individuals to addiction remain largely unknown. While animal models do not replicate the full spectrum of the human drug abuse syndrome, they have been used successfully to model drug-induced behavioral responses. Inbred mouse strains show extensive phenotypic variability for many drug-induced behaviors (Ruiz-Durantez et al. 2006; Seale and Carney 1991), and various techniques have been used to identify genetic differences that account for phenotypic variation (Boyle and Gill 2001; Bryant et al. 2012; Crabbe et al. 1999; Downing et al. 2006). However, these studies often result in the identification of large genomic regions containing hundreds of potential candidate genes, and identifying the causative genetic lesion has been difficult.

The chemical mutagen *N*-ethyl-*N*-nitrosourea (ENU) was first used in mice to induce heritable point mutations in the 1970s. In the 1990s, ENU mutagenesis experienced a resurgence as a method for assigning gene function and generating new mouse models for complex traits. ENU mutagenesis is a high-throughput approach for generating random heritable mutations in an unbiased manner, thereby allowing for the identification of phenotypes that have not yet been linked to a given gene. ENU-induced mutations have resulted in the identification of genes underlying complex phenotypes, including ataxia (Sharkey et al. 2009; Swanson et al. 2010; Xie et al. 2010), epilepsy (Frankel et al. 2009; Tokuda et al. 2011), and deafness (Grillet et al. 2009; Mackenzie et al. 2009; Parker et al. 2010; Schwander et al. 2009a, b). Mapping ENU-induced mutations for complex behavioral phenotypes has proven particularly difficult, but genes involved in locomotor activity have been identified (Furuse et al. 2010; Keays et al. 2004; Speca et al. 2010).

Here, we report the characterization of an ENU-induced mutant, *Highper*, that was discovered in a behavioral screen for open-field behavior. *Highper* mice exhibit novelty-induced hyperactivity, exaggerated locomotor response to psychostimulants, and increased sensitivity to the rewarding and reinforcing effects of cocaine. *Highper* mice also demonstrate a prolonged elevation of plasma corticosterone levels following acute restraint stress, indicating that *Highper* mice could model a link between stress and behavioral responses to psychostimulant drugs.

## Materials and methods

### Animals

C57BL/6J (B6) mice from the Jackson Laboratory (Bar Harbor, ME, USA) were bred at the in-house breeding colony at the Genomics Institute of the Novartis Research Foundation (GNF) and the University of North Carolina at Chapel Hill (UNC). Mice were maintained in an AAALAC-accredited, specific pathogen-free barrier colony in ventilated cages (Thoren Caging Systems (GNF), Hazelton, PA, USA or Tecniplast (UNC), Italy) on a 12-h light–dark cycle (lights on at 6:00 a.m. (GNF), 7:00 a.m. (UNC)). Mice were housed in groups of two to five in cages containing bedding (Bed-o-cob) and a cotton nestlet (GNF and UNC) or a PVC tube (UNC only). Irradiated food (Purina Pico rodent chow 20 (GNF) or Purina RMH 3000 (UNC), Purina, St. Louis, MO, USA) and water were provided ad libitum. Experimentally naïve mice were 59–70 days old at onset of testing. Due to shared animal room lighting constraints, behavioral tests were administered during the light part of the light/dark cycle (between 8:00 a.m. and 12:00 p.m.) except when otherwise noted. Locomotor behavior was tested in both light and dark cycles in a subset of mice to determine if *Highper* locomotor activity was dependent on time of day. All procedures were approved by the GNF, UNC, and Scripps Research Institute Institutional Animal Care and Use Committees following guidelines set forth by the National Institutes of Health (NIH) Guide for the Care and Use of Laboratory Animals.

### ENU mutagenesis and identification of the *Highper* mutant

ENU mutant mice were generated as previously described (Reijmers et al. 2006). Briefly, mutagenized G_0_ males were mated to B6 females, and G_1_ males were mated to B6 females. G_2_ daughters were mated to their G_1_ fathers to recover recessive mutations in G_3_ males that were screened for behavioral phenotypes. Outliers were ±2 standard deviations (SD) from the mean of the last 200 G_3_ mice screened.

A G_3_ outlier (>3 SD) from pedigree 262 was mated to a B6 female to establish the *Highper* line, and 70 F2s were open-field tested to determine heritability. F2s crossed to B6 mice produced F2N1s that were used to determine the mode of inheritance. Thereafter, the line was maintained by breeding presumed homozygotes.

### Drugs

Cocaine HCl (Sigma-Aldrich, St. Louis, MO) and methylphenidate HCl (MPD; Sigma-Aldrich, St. Louis, MO) were dissolved in 0.9 % saline. Based on the literature (Cunningham et al. 1999; Seale and Carney 1991) and data from our laboratory, 20 mg/kg cocaine induces moderate locomotor response in B6 and was used for initial locomotor activation studies. Multiple doses of cocaine (5, 20, 30, and 45 mg/kg) were used to assess dose response and self-administration (0.13, 0.25, 0.5, and 1.0 mg/kg). MPD was administered at 30 mg/kg. Ethanol was administered as a 20 % (*v*/*v*) solution at 0.75, 1.25, or 1.75 g/kg.

### Open-field behavior

The open-field apparatus (ENV-515-16, Med Associates, St. Albans, VT, USA) was a 43.2 × 43.2 × 33-cm arena consisting of a white Plexiglas floor and clear Plexiglas walls with infrared detection beams at 1-in. intervals on the *x*, *y*, and *z* axes that tracked the animals’ position and activity automatically throughout the experimental session. The apparatus was in a sound-attenuating chamber fitted with two overhead light fixtures containing 28-V lamps. Mice were moved to an anteroom for at least 1 h prior to testing, and animals were removed from their home cage, placed in the corner of the open-field arena, and allowed to freely explore the apparatus. Total distance traveled (centimeters) in the open-field arena was recorded in 2–5-min bins and scored in post-session analyses using commercially available software (Activity Monitor 5.1, Med Associates). Other measures of locomotor and exploratory behavior, including rearing, ambulatory episodes, and average velocity, were also assessed. The testing apparatus was cleaned with a 0.1 % bleach solution between test subjects.

### Locomotor response and habituation

Basal locomotor activity was recorded in a 10-min open-field session. To assess intersession and intrasession habituation effects on activity, mice were tested in one 10-min session for three consecutive days, and activity within and across days was analyzed.

### Circadian effects on locomotor activity

Locomotor activity in the open field was assessed in *Highper* (38 female and 19 male) and B6 (32 female and 29 male) mice in 10-min sessions during either the light (8:00 a.m. and 12:00 p.m.) or dark (8:00 p.m. and 12:00 a.m.) phase.

### Home cage activity

Home cage locomotor activity of *Highper* (11 females and 6 males) and B6 (eight females and six males) mice was assessed for 17 h (3:45 p.m.–8:45 a.m.). Mice were individually housed for 1 week prior to testing. On the day of testing, the entire mouse cage was placed into the open-field arena, and locomotor activity was recorded in 10-min bins. The sum of activity in the light phase (3:45–6:00 p.m., 6:00–8:45 a.m.) and the dark phase (6:00 p.m.–6:00 a.m.) was calculated. The effects of phase (light or dark), strain, and sex on locomotor activity were examined by ANOVA.

### Locomotor response to cocaine

To assess cocaine-induced acute locomotor response in a novel environment, naïve *Highper* (six male and ten female) and B6 (eight female and seven male) mice received an injection of 20 mg/kg cocaine or saline immediately prior to being placed in the open field for a 180-min session. The sum of the total distance traveled was calculated, and cocaine-induced activity was normalized to baseline activity by subtracting the within-strain average distance for saline-treated mice from individual distances for cocaine-treated mice.

Cocaine-induced locomotor activity was also measured in *Highper* (17 female and 14 male) and B6 (21 female and 19 male) mice after 3 days (intersession) and 3 h (intrasession) of habituation in the open field. On days 1–3, animals were given a sham saline injection and placed in the open-field arena for 10 min and then returned to their home cages. On day 4, animals were placed in the open-field arena for 60 min, given an injection of saline, and immediately returned to the arena for 120 min. The animals then received an injection of either saline or 20 mg/kg cocaine and were returned to the arena for 180 min.

### Locomotor response to methylphenidate

*Highper* (nine male and seven female) and B6 (11 male and 15 female) mice that had previously been tested in the open field for cocaine-induced locomotor behavior were tested 11–12 days later for locomotor response to 30 mg/kg MPD or saline. Mice were randomly assigned to receive either MPD or saline with regard to previous cocaine treatment so that treated and control groups were a mixture of mice that had received both cocaine and saline. The locomotor activity of mutant and control mice was assessed in a single 360-min open-field session, as described above. Total distance traveled during the 180-min interval following drug administration was calculated for each animal and normalized to baseline activity for each strain by subtracting the within-strain average distance for saline-treated mice from individual distances for MPD-treated mice.

### Alcohol-induced locomotor response and coordination

Alcohol-induced locomotor activity was assessed in *Highper* (40 male and 41 female) and B6 (83 male and 68 female) mice. On days 1–3, mice were injected with IP saline, and on day 4, mice received a 20 % (*v*/*v*) ethanol solution (0.75, 1.25, or 1.75 g/kg) or saline. Immediately following injection, mice were placed in the open field for 30 min. Distance over the test session was collected, and difference scores (day 4–day 3) were calculated. Immediately following each open-field session, animals were placed on the rotarod apparatus (Ugo Basile, Collegeville, PA, USA) for one trial. Revolutions per min (rpm) started at 3 rpm and progressively increased to 30 rpm during the 5-min trial. Latency to fall and latency to the first passive rotation were recorded. Animals that did not fall during the trial were assigned the maximum latency of 300 s. Rotarod performance was calculated as both the latency to fall and the latency to first passive rotation on day 4 (alcohol) as compared to day 3.

### Acute restraint and HPA activity

*Highper* (seven female and eight male) and B6 (seven female and seven male) mice were tested for restraint stress-induced hypothalamic–pituitary–adrenal (HPA) activation. Animals were restrained for 10 min in a Broome-style restrainer (Plas Labs, Inc., Lansing, MI, USA). Retro-orbital bleeds were performed immediately prior to restraint and 30 and 120 min post-restraint. Whole blood was centrifuged to isolate plasma, and corticosterone (CORT) levels were analyzed by competitive radioimmunoassay as per the manufacturer’s protocol (MP Biomedicals, Santa Ana, CA, USA). CORT was also measured during open-field testing. After 180 min in the arena, animals were given cocaine and returned for 180 min. Retro-orbital bleeds were performed on separate groups at 5 and 180 min (pre-cocaine) and 215 and 300 min (post-cocaine).

### Conditioned place preference

The rewarding properties of cocaine were tested in *Highper* (26 male and 23 female) and B6 (22 male and 19 female) mice using a three-chambered apparatus (MED-CPP-MSAT, Med Associates) as described in Eisener-Dorman et al. (2011). Briefly, mice were habituated on day 1, and time spent in either the black or white compartment was determined. Mice were randomly assigned to receive cocaine in either the black or white compartment (32 *Highper* and 30 B6). In a follow-up study, all mice received cocaine in the white compartment (17 *Highper* and 11 B6). On days 2, 4, 6, and 8, animals were given saline before placement in the unpaired compartment. On days 3, 5, 7, and 9, mice were given cocaine before placement in the drug-paired compartment. On day 10, mice could move freely between compartments, and percent time in the drug-paired compartment was compared to pre-conditioning. Activity on day 3 vs. day 2 was assessed as acute locomotor response, and activity on day 9 vs. day 3 was assessed as cocaine-induced locomotor sensitization.

### Cocaine self-administration

Cocaine self-administration testing was performed during the dark phase of the light/dark cycle. Male and female *Highper* and wild-type mice were catheterized using chronic jugular catheters (Pañeda et al. 2009) and trained to self-administer cocaine HCl (0.5 mg/kg, 15 mL infusion) in daily 1-h sessions using a fixed ratio (FR 1) schedule of reinforcement. A total of 32 *Highper* (16 male and 16 female) and 31 wild-type B6 (16 male and 15 male) successfully met self-administration criteria (>10 infusions per session, 70 % or more pressing on active lever, and variation of pressing on active lever less than 25 % across three consecutive days). These mice also had patent catheters, as verified by administering 0.02 ml of sodium brevital (5 mg/ml; Monarch Pharmaceuticals, Bristol, TN) 24–48 h after surgery and again at the completion of cocaine self-administration (i.e., prior to extinction sessions). Cocaine doses (0.125, 0.25, 0.5, and 1.0 mg/kg/infusion) were tested in daily 1-h sessions in a randomized order with the acquisition dose tested between each test dose. Dose effect curves were generated for each mouse strain. A subset of four to five mice per sex per genotype was subjected to progressive ratio (PR) testing. The PR procedure involved increasing the number of responses required to obtain a reinforcer until the animal failed to respond for 30 min. The last ratio completed was defined as the “breaking point.” The ratio progression followed the equation: [5e (injection number × 0.20)] − 5 (rounded to the nearest integer), such that the sequence of lever press requirements would begin: 1, 2, 4, 6, 9, 12, 15, 20, 25, 32, 40, 50, 62, 77, etc. Several doses (0.13, 0.25, 0.5, and 1.0 mg/kg) were tested with intervening FR 1 sessions. In the other 11–12 mice per sex per genotype the cocaine was removed and replaced with saline (i.e., the conditions of these trails were identical to the standard self-administration trials except that saline was substituted for cocaine), and lever pressing (i.e., extinction behavior) was determined over 10 days.

### Optomotor testing

Visual function was assessed in *Highper* (14 male and eight female) and B6 (eight male and nine female) mice using the optomotor assay (see Supplementary methods).

### Light/dark assay

*Highper* (14 male, eight female) and B6 (eight male, nine female) mice were tested for anxiety-like behavior in the light/dark assay (Crawley 1999) (see Supplementary methods).

### Spontaneous alternations (Y-maze)

Spontaneous alternation behavior, which measures spatial working memory, exploratory behavior, and response to novelty, was tested in *Highper* (14 male and 8 female) and B6 (eight male and nine female) mice (see Supplementary methods).

### Fear conditioning

*Highper* (6 male and 13 female) and B6 (6 male and 11 female) mice were tested for fear responses in the fear-conditioning assay (Med Associates, St. Albans, VT, USA) (see Supplementary methods).

### Statistical analyses

Data were analyzed using the SPSS statistical package (version 16.0 for Macintosh, SPSS, Chicago, IL, USA). Analysis of variance (ANOVA) tests were performed to analyze the effects of strain, sex, time point, treatment, and/or drug dose on the dependent variable. Dependent variables included total distance, activity difference score, CORT levels, percent time, movements, lever press count, and percent freezing. Mean differences were significant at *p* < 0.05. Error bars indicate SEM.

## Results

### The *Highper* mutation is recessive with reduced penetrance

**Fig. 1:**
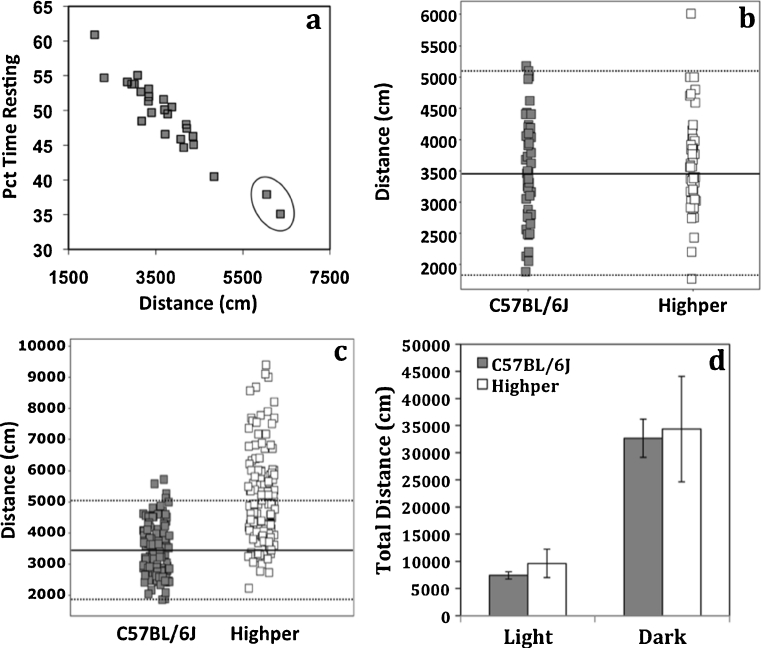
*Highper* mice are hyperactive in the open field. **a** Two G3 outliers (*circled*) from the same pedigree were identified as affected mutants. **b** Comparison of B6 wild-type and *Highper* F1 mice generated by crossing an affected F2 back to B6 to determine mode of inheritance. **c** Open-field data from presumed homozygous *Highper* mice at the F2–F8 generations and B6 controls show that the *Highper* mutation is likely a recessive mutation with approximately 50 % penetrance. *Solid* and *broken lines* represent the mean and two SD from the mean, respectively. **d** *Highper* mice do not exhibit hyperactivity in the home cage environment in either the dark or the light part of the circadian cycle. *Error bars* are SEM

Twenty-six G_3_ males from G_1_ pedigree 262 were tested in the open field, and two G_3_ outliers exhibited activity >3 SD from the mean of the last 200 mice screened (Fig. 1a). One of the male outliers was crossed to a B6 female, and open-field behavior was tested in 70 F2s. Eleven out of 70 F2s (14 %) exceeded an activity threshold defined as >2 standard deviations higher than the mean of a group of contemporaneously tested B6 mice, thereby confirming heritability. Mode of inheritance was tested by crossing affected F2s to B6 and testing 48 F2N1s. The *Highper* F2N1 population was not significantly different from 50 contemporaneously tested B6 mice (Fig. 1b); therefore, the *Highper* mutation did not fit a dominant mode of inheritance. The line was maintained by crossing presumed homozygotes, and activity was significantly elevated for all subsequent generations compared to contemporaneous controls (Fig. 1c). Based on the number of affected animals per generation (F3–F8) and the F2N1 data, the *Highper* mutation is recessive with reduced penetrance (Table 1).

**Table 1:** The *Highper* ENU mutant hyperactivity phenotype exhibits a recessive inheritance pattern with incomplete penetrance

Generation	Genotype (inferred)	Number tested	Expected number affected	Actual number affected	Penetrance (%)
G3	Mixed	26	3	2	66
F2	Mixed	70	18	11	61
F2N1	Heterozygous	48	0	3	NA
F3N1	Mixed	70	17	10	59
F3–F8	Heterozygous	127	127	61	48

Affected animals were identified by open-field activity scores >2 SD from the mean

### *Highper* mice are not hyperactive in the home cage

Home cage activity was analyzed to determine if the *Highper* phenotype was due to basal locomotor differences. An ANOVA for home cage activity showed a significant effect of phase (*F*_(1,61)_ = 16.8; *p* < 0.001) but no strain, sex, or interaction effects. All mice had increased activity during the dark phase, but no strain differences were observed indicating that *Highper* mice are not hyperactive in the home cage (Fig. 1d).

### *Highper* locomotor behavior is not light phase-dependent

To determine if the hyperactive phenotype of *Highper* mice was influenced by the time of day mice were tested, we measured open-field behavior in *Highper* during light and dark phases. A phase effect was observed (*F*_(1,117)_ = 8.6; *p* < 0.01) indicating that all mice showed significantly higher activity during the dark phase. Strain (*F*_(1,117)_ = 67.5; *p* < 0.001) and sex effects (*F*_(1,117)_ = 22.9; *p* < 0.001) were also observed. *Highper* mice exhibited increased activity compared to B6. A sex by strain interaction (*F*_(1,117)_ = 6.8; *p* < 0.05) was observed, and post hoc analysis indicated that *Highper* females exhibited higher activity than *Highper* males (*t*(55) = −4.0; *p* < 0.001). The sex difference was also significant in controls (*t*(59) = −2.3; *p* < 0.05). No strain by phase interaction was detected; therefore, time of day during testing did not alter strain activity differences (data not shown).

### *Highper* mice habituate to the open field

To examine whether the *Highper* phenotype was due to the novelty of the arena, we evaluated the effect of intersession habituation on locomotor activity in the open field in *Highper* (54 females and 42 males) and B6 (63 females and 57 males) mice over three consecutive days. ANOVA indicated strain (*F*_(1,215)_ = 90.3; *p* < 0.001), sex (*F*_(1,215)_ = 10.3; *p* < 0.01), and day effects (*F*_(2,215)_ = 26.5; *p* < 0.001). Females were more active than males, and *Highper* mice were more active than B6 for all 3 days. All mice were significantly less active on days 2 and 3 compared to day 1 (Supplementary Fig. 1). No interaction effects were observed; all mice habituated with repeated exposure, but *Highper* activity never diminished to B6 levels. A similar pattern was observed for other measures in the open field, including rearing (*F*_(2,215)_ = 20.9; *p* < 0.001), ambulatory episodes (*F*_(2,215)_ = 45.3; *p* < 0.001), and average velocity (*F*_(2,215)_ = 16.4; *p* < 0.001; data not shown).

### *Highper* mice exhibit increased locomotor response to cocaine

Test-naïve *Highper* mice were assessed for locomotor response to an acute cocaine dose. *Highper* mice showed a greater cocaine-induced locomotor response compared to B6, but this difference was not significant after subtracting mean baseline activity (saline) from cocaine-induced activity for each animal. No significant strain (*F*_(1,15)_ = 0.37; *p* > 0.05), sex (*F*_(1,15)_ = 0.33; *p* > 0.05), or strain by sex interaction effects (*F*_(1,15)_ = 0.00; *p* > 0.05) were detected (Supplementary Fig. 2).

**Fig. 2:**
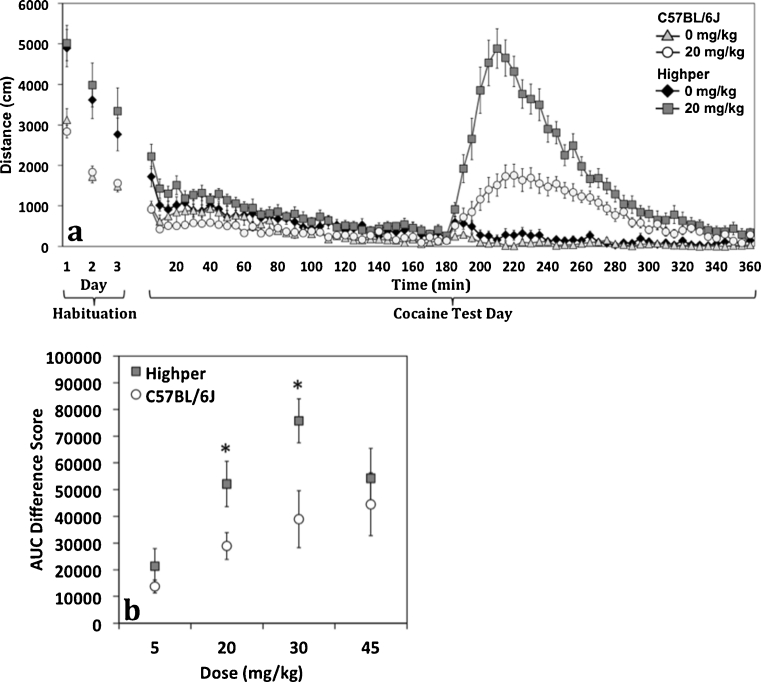
Habituation alters open-field locomotor response to an acute dose of 20 mg/kg cocaine in *Highper* mice. **a** Cocaine-induced psychomotor response is significantly greater in *Highper* mice when the drug is administered following habituation to the open-field arena. **b** Dose response to four doses of cocaine in *Highper* and B6 mice. Cocaine-induced locomotion was normalized to saline-induced locomotion to correct for baseline activity differences. *Error bars* are SEM. *p < 0.05

To mitigate the effects of high baseline activity in *Highper* mice, we established a protocol for 3 days (intersession) and 3 h (intrasession) of habituation prior to cocaine administration. Post-habituation, all mice exhibited similar activity immediately preceding cocaine administration (*F*_(1,37)_ = 3.9; *p* > 0.05). We examined total activity for the 180 min post-cocaine administration, normalized for saline-induced activity and identified a strain effect (*F*_(1,37)_ = 19.3; *p* < 0.001). When cocaine was administered after habituation, *Highper* mice showed a significantly higher locomotor response (Fig. 2a). No sex (*F*_(1,37)_ = 2.3; *p* > 0.05) or sex by strain interaction effects (*F*_(1,37)_ = 1.6; *p* > 0.05) were detected. *Highper* mice also make more ambulatory episodes in comparison to B6 following cocaine administration (*F*_(1,71)_ = 5.3; *p* < 0.05). The same difference was not observed for either rearing behavior (*F*_(1,71)_ = 0.21; *p* > 0.05) or average velocity (*F*_(1,71)_ = 0.18; *p* > 0.05; Supplementary Fig. 3).

Cocaine dose response was also assessed in *Highper* and B6 controls. Cocaine-induced locomotor activity (normalized to saline-treated controls) was analyzed by ANOVA, and strain (*F*_(1,65)_ = 10.48; *p* < 0.01) and dose (*F*_(3,65)_ = 7.06; *p* < 0.001), but no strain by dose interaction effects (*F*_(3,65)_ = 0.99; *p* > 0.05) were detected. Cocaine-induced locomotor response was greater in *Highper* than in B6 mice at 20 and 30 mg/kg but not at 5 (*F*_(1,15)_ = 1.9; *p* > 0.05) or 45 mg/kg doses (*F*_(1,15)_ = 0.36; *p* > 0.05; Fig. 2b). No sex differences were observed (*F*_(1,65)_ = 0.57; *p* > 0.05).

### Drug-induced hyperactivity in *Highper* mice is not cocaine-specific

We tested *Highper* mice for sensitivity to MPD. Because the mice were previously treated with a single dose of cocaine or saline to assess cocaine-induced locomotor activation, we tested the effect of previous treatment on MPD response. No previous treatment effect on MPD response was observed (*F*_(1,41)_ = 0.1; *p* > 0.05) nor were any interaction effects observed between prior cocaine exposure and strain (*F*_(1,41)_ = 0.91; *p* > 0.05) or prior exposure, strain, and MPD dose (F_(1,41)_ = 0.98; *p* > 0.05). Therefore, prior cocaine exposure did not affect MPD response. Strain (*F*_(1,41)_ = 15.0; *p* < 0.01) and dose effects (*F*_(1,41)_ = 138.5; *p* < 0.001) and a strain by dose interaction (*F*_(1,41)_ = 9.8; *p* < 0.01) were observed. All mice showed MPD-induced locomotor activation, but the *Highper* activity was significantly higher than B6 activity (Supplementary Fig. 4).

We tested the *Highper* locomotor response to ethanol. Dose (*F*_(3,231)_ = 9.1; *p* < 0.001), strain (*F*_(1,231)_ = 32.6; *p* < 0.001), and strain by dose interaction effects were observed (*F*_(3,231)_ = 3.4; *p* < 0.05). Post hoc analysis indicated that *Highper* mice showed higher locomotor responses to the 0.75 (*p* < 0.01) and 1.25 g/kg (*p* < 0.001) doses but not to the 1.75 g/kg dose (Supplementary Fig. 5). No sex differences were observed (*F*_(1,231)_ = 3.4; *p* > 0.05).

Locomotor coordination on the rotarod following exposure to the same doses of alcohol was also assessed in *Highper* and B6 mice. ANOVA of latency to fall from rotarod (difference scores) showed a dose effect (*F*_(3,199)_ = 10.91; *p* < 0.001). Post hoc analysis showed a significant difference in response to 1.25 (*p* < 0.01) and 1.75 g/kg (*p* < 0.001) compared to saline and between 0.75 and 1.25 g/kg (*p* < 0.05) and 0.75 and 1.75 g/kg (*p* < 0.001). No sex (*F*_(1,199)_ = 1.55; *p* > 0.05), strain (*F*_(1,199)_ = 1.23; *p* > 0.05), sex by strain (*F*_(1,199)_ = 0.27; *p* > 0.05), sex by dose (*F*_(3,199)_ = 1.78; *p* > 0.05), or strain by dose interaction effects (*F*_(3,199)_ = 0.30; *p* > 0.05) were identified (data not shown).

### *Highper* mice exhibit greater cocaine-induced CPP

Mice spent a similar amount of time in the black and white chambers on day 1 indicating that the conditioned place preference (CPP) apparatus was unbiased (*t*(178) = 0.87; *p* > 0.05, data not shown). However, ten animals (six *Highper*, four B6) spent significantly more time in the black or white chamber (>3 or <3 SD from group mean) and were removed from CPP analysis. Day 1 activity differed by strain (*F*_(1,173)_ = 15.7; *p* < 0.001); *Highper* mice were more active than B6.

**Fig. 3:**
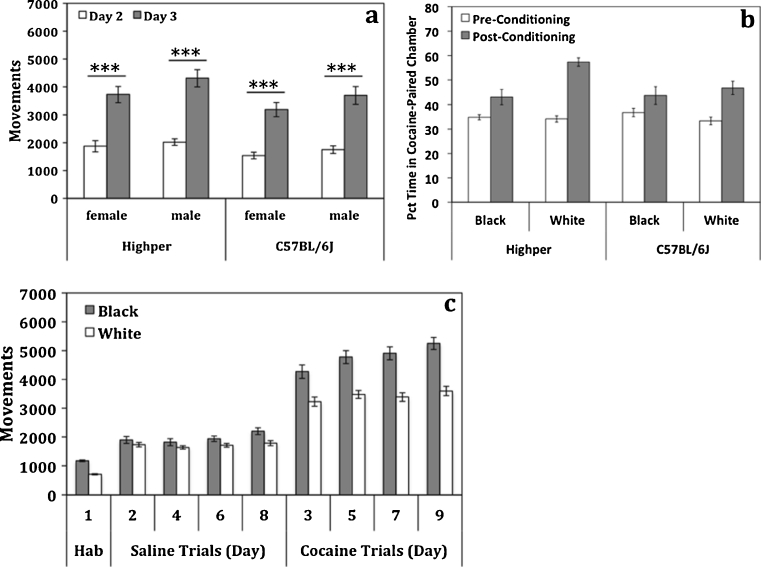
*Highper* mice exhibit increased context-specific place preference and acute locomotor response to 20 mg/kg cocaine. **a** Acute locomotor response was determined by comparing activity in the conditioned place preference apparatus on day 3 (first cocaine treatment) and day 2 (saline). **b** Cocaine was administered either in the *black* or *white* CPP chamber. Place preference was significantly higher in *Highper* mice when cocaine was paired with the white chamber but not the black chamber. **c** Locomotor activity was higher in the black chamber of the place preference apparatus during habituation (day 1) and conditioning (days 2–9). Chamber-specific activity differences were present in both saline- and cocaine-treated animals. *Error bars* are SEM. *p < 0.05, **p < 0.01, and ***p < 0.001

All mice showed increased activity after the first cocaine treatment (*F*_(1,178)_ = 209.2; *p* < 0.001), but cocaine-induced locomotor activation was higher in *Highper* mice (Fig. 3a). Activity was significantly greater on day 9, after the final cocaine treatment, than on day 3 (*p* < 0.01) in both *Highper* and B6 mice. However, this difference was dependent on the chamber in which cocaine was administered (*p* < 0.001). When each chamber was considered separately, sensitization occurred only in the black chamber (*p* < 0.05) and only in B6 mice (*p* < 0.05) (Supplementary Fig. 6).

ANOVA indicated that conditioning chamber affected CPP development (*F*_(1,55)_ = 7.3; *p* < 0.01); mice conditioned in the white chamber showed significantly higher CPP. In a follow-up study, mice were conditioned in the white chamber only, and *Highper* mice had significantly higher CPP (*F*_(1,23)_ = 6.9; *p* < 0.05; Fig. 3b). All mice exhibited significantly higher activity in the black chamber compared to the white chamber (*F*_(1,173)_ = 176.1; *p* < 0.001). This activity difference persisted throughout saline (*F*_(1,244)_ = 16.6; *p* < 0.001) and cocaine conditioning (*F*_(1,247)_ = 96.7; *p* < 0.001; Fig. 3c).

### *Highper* mice show increased responding for lower doses of cocaine

**Fig. 4:**
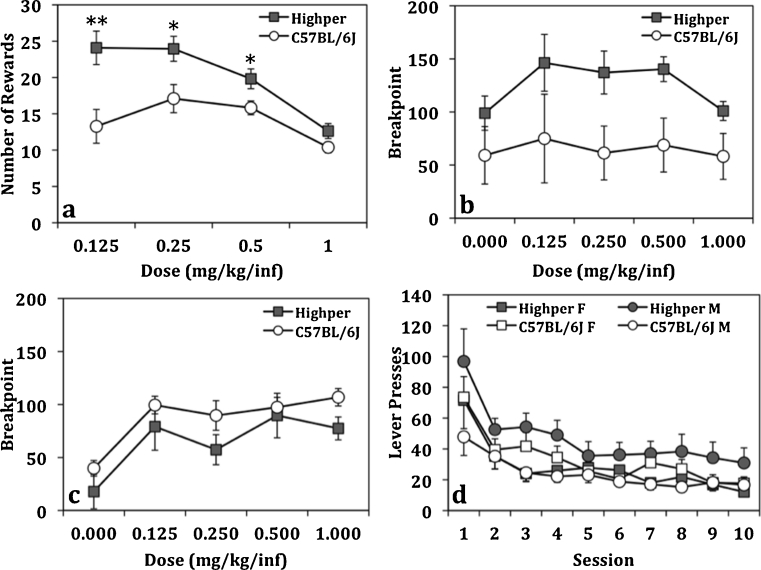
*Highper* mice are sensitive to the rewarding effects of cocaine. **a** *Highper* mice self-administered more cocaine at the lower doses (0.125, 0.25, and 0.5 mg/kg/inf). **b**, **c** Female, but not male, *Highper* mice worked harder for cocaine in a progressive ratio schedule of self-administration. **d** All mice show extinction of cocaine-seeking behavior across ten sessions, whereas *Highper* males had higher levels of responding relative to B6 males. *Error bars* are SEM

Analysis of the number of cocaine rewards obtained during dose response for the self-administration study identified sex (*F*_(1,59)_ = 4.5; *p* < 0.05), dose (*F*_(3,177)_ = 16.9; *p* < 0.001), strain (*F*_(1,59)_ = 14.1; *p* < 0.001), and strain by dose interaction (*F*_(3,177)_ = 4.1; *p* < 0.01) effects. All mice showed decreased number of rewards as dose increased, and males administered more rewards than females. *Highper* mice administered more rewards at the two lowest doses (Fig. 4a). This result was not due to general activity differences because *Highper* mice responded more on the active lever (*F*_(1,59)_ = 7.5;c*p* < 0.01) than the inactive lever (*F*_(1,59)_ = 0.4;c*p* > 0.05; Supplementary Fig. 7). A subset of mice was used to examine the motivation of *Highper* mice using a PR schedule. There were no significant strain effects on progressive ratio breakpoints. *Highper* females had higher breakpoints than their B6 counterparts (*F*_(1,7)_ = 4.5; *p* < 0.05), which was not observed in males (Fig. 4b, c). All mice showed lever pressing extinction (*F*_(9,351)_ = 25.5; *p* < 0.0001), but there was no overall strain difference. A strain by sex interaction (*F*_(1,39)_ = 4.6; *p* < 0.05) revealed that *Highper* males had higher lever responding than their B6 counterparts (*F*_(1,21)_ = 5.9; *p* < 0.05; Fig. 4d).

### *Highper* mice show prolonged stress-induced HPA activation

**Fig. 5:**
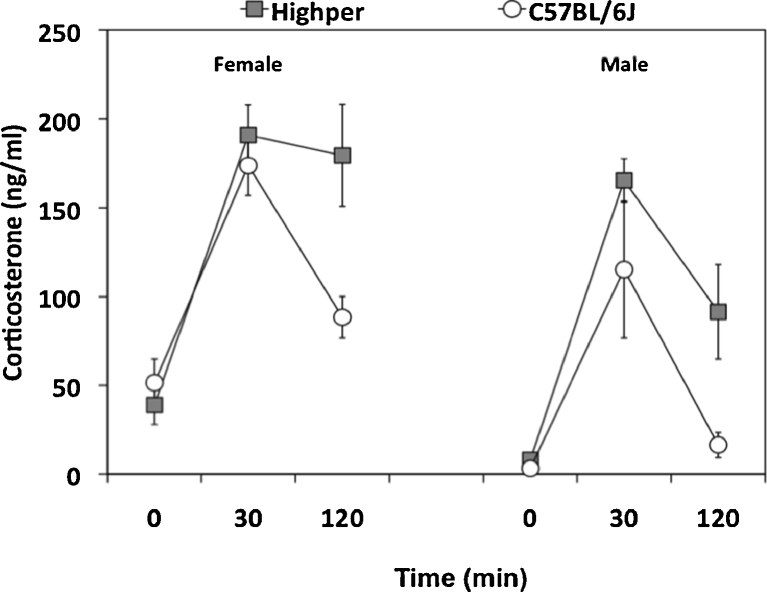
*Highper* mice have prolonged HPA activation in response to restraint-induced stress. Corticosterone levels (nanograms per milliliter) were assessed at baseline and 30- and 120-min post-restraint for female (**a**) and male mice (**b**). *Error bars* are SEM. *p < 0.05

Previous studies identified a correlation between locomotor response to novelty and prolonged HPA stress reactivity (Piazza et al. 1991). We tested acute restraint stress in *Highper* mice and identified strain (*F*_(1,85)_ = 12.1; *p* < 0.01), sex (*F*_(1,85)_ = 25.0; *p* < 0.001), and time effects (*F*_(2,85)_ = 55.3; *p* < 0.001) and a strain by time interaction (*F*_(1,85)_ = 5.5; *p* < 0.01). CORT levels were higher in females and in *Highper* mice. In both strains, CORT increased significantly from 0 to 30 min post-restraint. At 120 min, CORT levels remained elevated in *Highper* mice but decreased to basal levels in B6 (Fig. 5a, b).

### The abnormal cocaine-induced locomotor response exhibited by *Highper* mice is not related to CORT levels during open-field testing

To determine if the abnormal drug-induced locomotor response exhibited by *Highper* mice is related to prolonged HPA activation, cocaine was administered prior to open-field testing, and CORT levels were assessed at four different timepoints. No strain effect was observed indicating that CORT release during open-field testing was similar for all mice. However, females had higher CORT levels (*F*_(1,91)_ = 40.8; *p* < 0.001). CORT levels were similar at 5 and 180 min (pre-cocaine; *p* > 0.05) but increased significantly at 215 and 330 min (post-cocaine; *p* < 0.001). This effect was more prominent in females (*F*_(3,46)_ = 43.0; *p* < 0.001) than in males (*F*_(3,46)_ = 6.2; *p* < 0.01; Supplementary Fig. 8).

### *Highper* mice do not exhibit anxiety-like behavior

We tested *Highper* mice in the light/dark task to evaluate anxiety-like behavior. An ANOVA of the amount of time spent in the light side showed no strain (*F*_(1,38)_ = 3.24; *p* > 0.05), sex (*F*_(1,38)_ = 0.01; *p* > 0.05), or strain by sex interaction effects (*F*_(1,38)_ = 0.91; *p* > 0.05) suggesting that *Highper* mice do not differ for anxiety-like behavior in comparison to B6 (data not shown). *Highper* mice exhibited increased light/dark transitions (*F*_(1,38)_ = 25.1; *p* < 0.001) that is likely due to their novelty-induced hyperactivity phenotype (Supplementary Fig. 9).

### *Highper* mice do not exhibit visual deficits

Optomotor testing was performed to identify visual tracking deficiencies. ANOVA analysis showed no strain (*F*_(1,38)_ = 3.0; *p* > 0.05), sex (*F*_(1,38)_ = 0; *p* > 0.05), or strain by sex interaction effects (*F*_(1,38)_ = 0.64; *p* > 0.05) indicating that *Highper* mice have no obvious visual impairments (Supplementary Fig. 10).

### *Highper* mice show no deficits in spontaneous alternation

Spontaneous alternations were measured to assess spatial memory, exploratory behavior, and response to novelty in *Highper* mice. An ANOVA for the number of arm entries in the Y-maze revealed a strain effect (*F*_(1,38)_ = 42.1; *p* < 0.001) but no sex effect (*F*_(1,38)_ = 2.46; *p* > 0.05) and no strain by sex interaction (*F*_(1,38)_ = 3.4; *p* > 0.05) indicating that *Highper* mice exhibited increased exploratory behavior (data not shown). An ANOVA for the percent of spontaneous alternation observed showed no strain (*F*_(1,38)_ = 0.6; *p* > 0.05), sex (*F*_(1,38)_ = 0.90; *p* > 0.05), or strain by sex interaction effects (*F*_(1,38)_ = 1.24; *p* > 0.05), which suggests that spatial working memory is similar for *Highper* and B6 mice (Supplementary Fig. 11).

### *Highper* mice show decreased freezing behavior in the fear conditioning assay

For day 1 baseline freezing, a strain effect (*F*_(1,35)_ = 10.0; *p* < 0.01) but no sex (*F*_(1,35)_ = 2.0; *p* > 0.05) or strain by sex interaction effects (*F*_(1,35)_ = 0.20; *p* > 0.05) were observed prior to tone–shock training. *Highper* mice froze significantly less than B6.

Freezing during the day 1 intertrial interval after each tone–shock pairing was analyzed by ANOVA; significant strain (*F*_(1,179)_ = 51.9; *p* < 0.001), sex (*F*_(1,179)_ = 4.0; *p* < 0.05), and interval effects (*F*_(3,179)_ = 6.2; *p* < 0.01) were observed. Females froze more than males, and *Highper* mice froze significantly less than B6. Freezing increased significantly across intervals with each successive tone–shock pairing. On day 2, *Highper* mice froze less during the first 3 min of the session (*F*_(1,35)_ = 4.2; *p* < 0.05). Analysis of freezing during intertrial intervals on day 2 yielded results similar to day 1; strain (*F*_(1,143)_ = 17.3; *p* < 0.001), sex (*F*_(1,143)_ = 5.4; *p* < 0.05), and trial effects (*F*_(3,143)_ = 5.4; *p* < 0.01) were observed. As on day 1, *Highper* mice froze significantly less than B6, and females froze significantly more than males. Freezing at the last intertrial interval was significantly higher than freezing at the first intertrial interval indicating that fear learning had occurred. Freezing after day 2 conditioning was higher than after day 1 (*t*(70) = −2.7; *p* < 0.01). This effect did not differ between strains, but overall the strains differed in freezing at the end of both days.

Our test of cued fear memory revealed no significant difference between day 3 baseline freezing in the new context and day 1 baseline freezing (*F*_(1,70)_ = 1.8; *p* > 0.05); thus, animals did not show generalized freezing. No significant strain (*F*_(1,70)_ = 1.5; *p* > 0.05) or sex effects (*F*_(1,70)_ = 4.1; *p* > 0.05) were observed for day 3 baseline freezing. All mice exhibited increased freezing to tones on day 3 (*F*_(2,104)_ = 12.0; *p* < 0.001). Freezing increased in response to tone 1 (*p* < 0.001) and tone 2 (*p* < 0.001) relative to baseline freezing, but freezing did not differ between the two tones (Supplementary Fig. 12).

## Discussion

The *Highper* mutant displays hyperactivity in novel environments, exaggerated locomotor response to psychostimulants, increased sensitivity to the rewarding properties of cocaine, and prolonged stress-induced HPA activation. The absence of sensory and learning and memory deficits removes these potential confounds from the interpretation of our results. Based on the convergence of drug response, reward and reinforcement differences, as well as alterations in HPA reactivity, we propose that *Highper* mice may be useful for studying the intersection between drug reward and stress response pathways.

The increased locomotor activity in *Highper* mice appears to be novelty-specific because home cage activity did not differ between mutant and wild-type mice. However, *Highper* mice were more active than controls in the open field over multiple testing days; thus, *Highper* mice may exhibit general hyperactivity in test environments. We tested the hypothesis that response to novelty was due to differences in stress response, like those induced by acute restraint; however, CORT levels did not differ between *Highper* and B6 mice during open-field testing. Differing HPA response to diverse stressors (e.g., open field vs. restraint) is not surprising and has been reported elsewhere (Bowers et al. 2008).

Increased locomotor response to novelty, as observed in *Highper* mice, correlates with increased psychomotor effects of cocaine in mice (Brabant et al. 2005) and for a variety of psychostimulants in high-responder (HR) and low-responder rats (Belin et al. 2011; Kiyatkin 1992; Deroche et al. 1993; Piazza et al. 1989, 1990). The extent to which novelty-induced locomotor activity behavior translates to more active models of drug seeking or drug taking (i.e., CPP or CSA) has been debated. Whereas some studies have identified a correlation between locomotor response to novelty and drug self-administration (Piazza et al. 1989, 1990; Thomsen and Caine 2011), others have not (Gong et al. 1996; Kosten et al. 2007). In addition, correlations between novelty-induced activity and CPP are often not observed (Erb and Parker 1994; Gong et al. 1996), but these varying results could be due to dose effects because several studies in mice using lower doses of cocaine (4–5 mg/kg) have reported a significant negative correlation (Brabant et al. 2005; Shimosato and Watanabe 2003).

Other studies have reported an inverse correlation between locomotor activity and CPP (Gremel and Cunningham 2007; Cunningham et al. 1999; Vezina and Stewart 1987; Neisewander et al. 1990; Sora et al. 2001). It has been suggested that high activity levels may interfere with the subject’s ability to effectively process its surroundings, thereby reducing associative learning and resulting in decreased CPP. Significantly greater CPP was observed in *Highper* mice given cocaine in the white compartment in which cocaine-induced locomotor activation was significantly decreased. We theorize that the reduced activity engendered by the white environment caused more robust expression of CPP in the *Highper* mutant, allowing us to observe true strain differences. This theory is supported by our observation that *Highper* mice show a more robust response to the psychomotor effects of cocaine after intrasession habituation to the arena that reduces basal activity to wild-type levels. Thus, the overall hyperactivity of *Highper* mice in novel and/or testing situations may mask the stimulatory and rewarding effects of psychostimulants.

B6 and *Highper* mice show sensitization upon repeated cocaine administration but only in mice trained in the black compartment, indicating that suppression of activity in the white compartment may also alter sensitization. Increased locomotor response follows the second cocaine treatment in *Highper* mice and is maintained throughout conditioning but may be limited by a ceiling effect due to the small CPP chamber size. In B6 mice, a gradual increase in locomotor response occurs across all 4 days of cocaine administration. We previously reported that CPP chamber characteristics are critical in the expression of cocaine sensitization in BALB/cByJ (Eisener-Dorman et al. 2011), and increased sensitization to psychostimulants has been observed in rats when drugs are administered in novel rather than home environments; therefore, sensitization may be increased in environments associated with greater activity. The causes of context-specific sensitization are not yet clear, but the HPA axis and dopamine neurotransmission have been implicated (Badiani et al. 1995; Goeders 2002a; Williams and Adinoff 2008).

The primary finding in the cocaine self-administration study was increased responding by *Highper* mice for cocaine at the lowest doses tested with no overall strain differences in inactive lever responding, progressive ratio responding, or responding during extinction. Importantly, all mice showed an increase in lever pressing in the initial extinction trials, suggesting that they were indeed lever pressing for reward and not simply falling into habitual lever pressing behavior. When the sexes were analyzed separately, female *Highper* mice had higher progressive ratio breakpoints than female B6 mice, and male *Highper* mice had higher active lever presses during extinction than male B6 mice. It should be noted that a dose effect curve was not observed in either B6 or *Highper* mice during PR tests. While PR dose effect curves are consistently observed in rats, they can be more difficult to capture in mice. In fact, it is more common to see single-dose PR tests in the mouse literature. Depending on the background strain and the specific methods used (acquisition protocol, dose order, intervening day treatment), dose effect curves, albeit not necessarily inverted U-shaped ones, have been observed using a between-subjects design or when the lowest dose is tested last (Alsio et al. 2011; Schmidt et al. 2011; Sorensen et al. 2012). However, under other experimental conditions, dose effect curves have not been observed (Ozburn et al. 2012).

Considered together, the self-administration and place preference data are consistent with the hypothesis that *Highper* mice are more sensitive to the rewarding effects of cocaine. However, it should be noted that the place preference assay was only performed with a single dose of cocaine and, therefore, is not generalizable over a wider dose range.

*Highper* mice subjected to acute restraint stress exhibit a prolonged HPA response similar to that of HR rats. The HPA axis has previously been implicated in cocaine behaviors (Piazza and Le Moal 1998). Acute cocaine administration increases circulating CORT levels (Borowsky and Kuhn 1991; Levy et al. 1991; Mello and Mendelson 1997), which increases sensitivity to lower doses of cocaine (Goeders 2002a, b; Goeders and Guerin 1994; Mantsch et al. 1998). Adrenalectomy reduces the psychomotor properties of cocaine, and supplementation of CORT to basal levels reverses the effect (Marinelli et al. 1994). In addition, stressors enhance the acquisition of self-administration (Goeders 2002b; Piazza and Le Moal 1998). These data lead to the hypothesis that increased HPA reactivity renders *Highper* mice more sensitive to the activating effects of psychostimulants. However, *Highper* mice do not differ in basal CORT or in circulating CORT following cocaine exposure in the open field, so HPA activation during open-field testing does not explain their locomotor response to cocaine.

The HPA axis also interacts with mesolimbic dopaminergic neurons in brain regions that have been implicated in drug reward (Piazza and Le Moal 1996, 1998). Glucocorticoid hormones increase dopamine release in the nucleus accumbens and sensitivity to the psychomotor effects of psychostimulants (Marinelli et al. 1994). *Highper* and B6 mice may differ in some aspect of the mesolimbic dopaminergic system, and further studies are warranted. Early life stressors, including prenatal stress and deficits in maternal care, are also implicated in increased sensitivity to psychostimulants (Kippin et al. 2008; Li et al. 2003), and we cannot eliminate the possibility that *Highper* mothers experience higher HPA activity during gestation or differ in the maternal care provided to their offspring.

A mutation affecting the dopaminergic pathway is a plausible explanation for the observed phenotypes given that *Highper* mice show increased locomotor activity, exaggerated locomotor responses to both cocaine and ethanol, and increased sensitivity to the rewarding and reinforcing effects of cocaine—all behaviors that are mediated by the mesolimbic dopamine and the brain reward pathways. Interestingly, cocaine- and ethanol-induced locomotor activations are genetically related in ethanol FAST and SLOW lines selectively bred for sensitivity to ethanol (Meyer et al. 2009). Injection of both cocaine and ethanol results in greater levels of dopamine in the nucleus accumbens of FAST mice (Meyer et al. 2009). The quantification of brain dopamine levels in *Highper* mice during open-field testing and in response to psychostimulant administration is the next logical step in determining if increased dopamine is related to the locomotor and drug-related behaviors.

The identification of the causative gene is a crucial step towards understanding the spectrum of *Highper* mutant phenotypes. Previous gene mapping efforts for ENU behavioral mutants have been slowed by limitations in the tools available for gene identification. However, recent advances in SNP discovery and the emergence of accessible sequencing technology provide the tools necessary to identify ENU-induced mutations. Phenotypic characterization of mouse models of cocaine-induced locomotor activation, such as the *Highper* mutant, will expand the existing knowledge of the neurobiological pathways driving initial sensitivity to cocaine. Consequently, identification of the causative mutation in *Highper* will lead to the discovery of genes or gene pathways that may influence individual susceptibility to addiction.

## Electronic supplementary material

Below is the link to the electronic supplementary material.

ESM 1 (PDF 629 kb)
