# Supplementary material for: Characterization of Highper, an ENU-induced mouse mutant with abnormal psychostimulant and stress responses
Source: Psychopharmacology (Berl). 2012 Sep 5;225(2):407–19. doi: 10.1007/s00213-012-2827-5 (PMC3536991; doi:10.1007/s00213-012-2827-5)
Supplement: Supplementary file 1 — (PDF 629 kb) [file 213_2012_2827_MOESM1_ESM.pdf]

Supplementary Materials and Methods

Optomotor testing

The optomotor platform is stationary, elevated and surrounded by a drum with black and white striped walls. The mouse was placed on the platform to habituate for 1 min and then the drum was rotated at 2 revolutions per min (rpm) in one direction for 1 min, stopped for 30 sec, and then rotated in the other direction for 1 min. The number of head tracks (15 degree movements at speed of drum) was recorded. Mice with intact vision track 6–20 times, whereas blind mice do not track at all.

Light/dark (LD) assay

The LD apparatus is a rectangular box made of Plexiglas (Ridout Plastics, San Diego, CA) and partitioned into two environments—one dark (14.5 x 27 x 26.5 cm, 8-16 lux) and the other highly illuminated (28.5 x 27 x 26.5 cm, 400-600 lux) by a 60W light source located above it. The compartments were connected by an opening (7.5 x 7.5 cm) located at floor level in the center of the partition. Mouse behavior was videotaped, and a trained technician blind to genotype analyzed time spent in the light compartment and numbers of transitions from the dark into the light compartment (Crawley and Goodwin 1980). Mice are placed in the dark compartment to start the 5-minute test.

Spontaneous alternations (Y-maze)

Spontaneous alternation behavior, a measure of spatial working memory, exploratory behavior and responsiveness to novelty (Hughes 2004; Lalonde 2002), was tested in *Highper* (14 male, 8 female) and B6 (8 male, 9 female) mice. Each mouse received a 5-min trial. Arm choices and arm entries were recorded. Spontaneous alternation is the ratio of arm choices differing from the previous two choices to the total number of arm entries.

Fear conditioning

Conditioned and cued fear behavior was measured as described by Reijmers (Reijmers et al. 2006). On Day 1, baseline freezing was recorded for 3 min followed by four tone-shock pairings comprised of a 20-sec tone (85 dB, 2800 Hz) that ended simultaneously with a 2-sec shock (0.75 mA). The four tone-shock pairings were separated by 1-min intertrial intervals and the mice remained in the chamber for 1 min after the final tone-shock presentation prior to returning to the home cage. Day 2 repeated the

training trial from Day 1, and freezing was recorded for the first 3 min as a measure of 1-day contextual freezing. On Day 3, mice were tested in the same apparatus, but all of the contextual cues in the chamber were changed – a V-shaped wall was present along with a smooth floor covered with bedding. Baseline freezing was recorded for 3 min to measure generalized freezing behavior in the new context. Two 20-sec tones were then presented, separated by a 1-min intertrial interval, and freezing that occurred during the tone was recorded.

Supplementary Figure Legends

Supplementary Fig. 1 *Highper* mice exhibit both inter- and intrasession habituation in the open-field arena. Error bars are SEM.

Supplementary Fig. 2 Experimentally naïve *Highper* mice exhibit slightly higher locomotor response to 20 mg/kg cocaine over a 180-min session. After controlling for baseline activity, the difference was not significant.

Supplementary Fig. 3 Increased rearing behavior (a) and average velocity (b) in response to 20 mg/kg cocaine in the open field in *Highper* and B6 mice. Sexes combined. Error bars are SEM. ***p<0.001.

Supplementary Fig. 4 *Highper* mice exhibit an increased psychomotor response to 30 mg/kg methylphenidate in the open-field arena. Error bars are SEM.

Supplementary Fig. 5 *Highper* mice exhibit an increased acute locomotor response to alcohol at 0.75 and 1.25 g/kg doses. Acute locomotor response was determined by normalizing open-field activity on Day 4 (alcohol-treated) to Day 3 (saline-treated). Error bars are SEM.

Supplementary Fig. 6 Locomotor sensitization to 20 mg/kg cocaine. Sensitization was assessed by comparing activity across conditioning days when cocaine was administered (Days 3, 5, 7 and 9). Error bars are SEM.

Supplementary Fig. 7 *Highper* mice make more lever presses on the active lever and do not differ from B6 for inactive lever presses. Error bars are SEM. *p<0.05, **p<0.01.

Supplementary Fig. 8 The hyperactive phenotype of *Highper* mice in the open field is not caused by abnormal CORT release. CORT levels (ng/ml) were assessed at 5, 180, 215 or 300 min in the open-field arena. CORT levels increased after cocaine administration at 180-min and differed significantly between males and females. Error bars are SEM. Asterisks depict differences between males and females. *p<0.05, ***p<0.001

Supplementary Fig. 9 Number of transitions (a) and percent time in the lighted compartment (b) in the light/dark assay. Error bars are SEM. ***p<0.001.

Supplementary Fig. 10 Number of head tracks in the optomotor assay. Error bars are SEM.

Supplementary Fig. 11 Alternations (a), spontaneous alternations (b) and number of arm entries (c) in the Y-maze. Error bars are SEM. ***p<0.001.

Supplementary Fig. 12 *Highper* mice show relatively normal fear-based learning and memory, although the development of freezing behavior is slower in mutant mice. Percent freezing is shown at baseline and inter-tone intervals (ITI) for the first two days of testing. Contextual and

cued testing occurred on Days 2 and 3, respectively. Error bars are SEM. Asterisk represent strain differences. *$p<0.05$, **$p<0.01$

Supplementary Figure 1

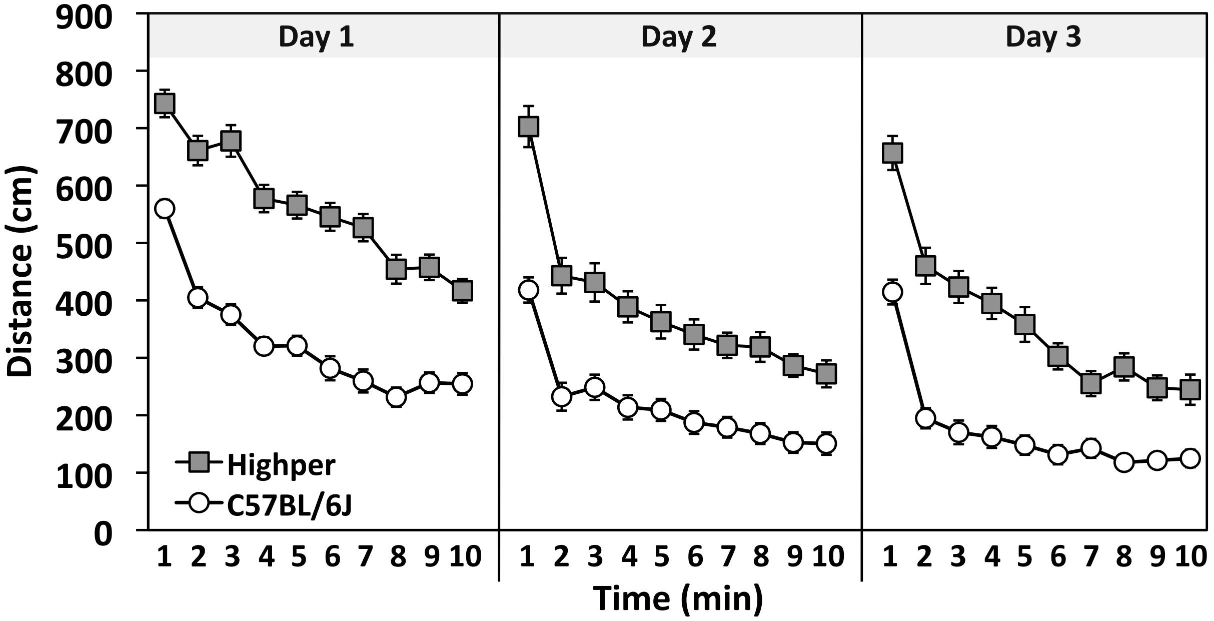

Supplementary Figure 2

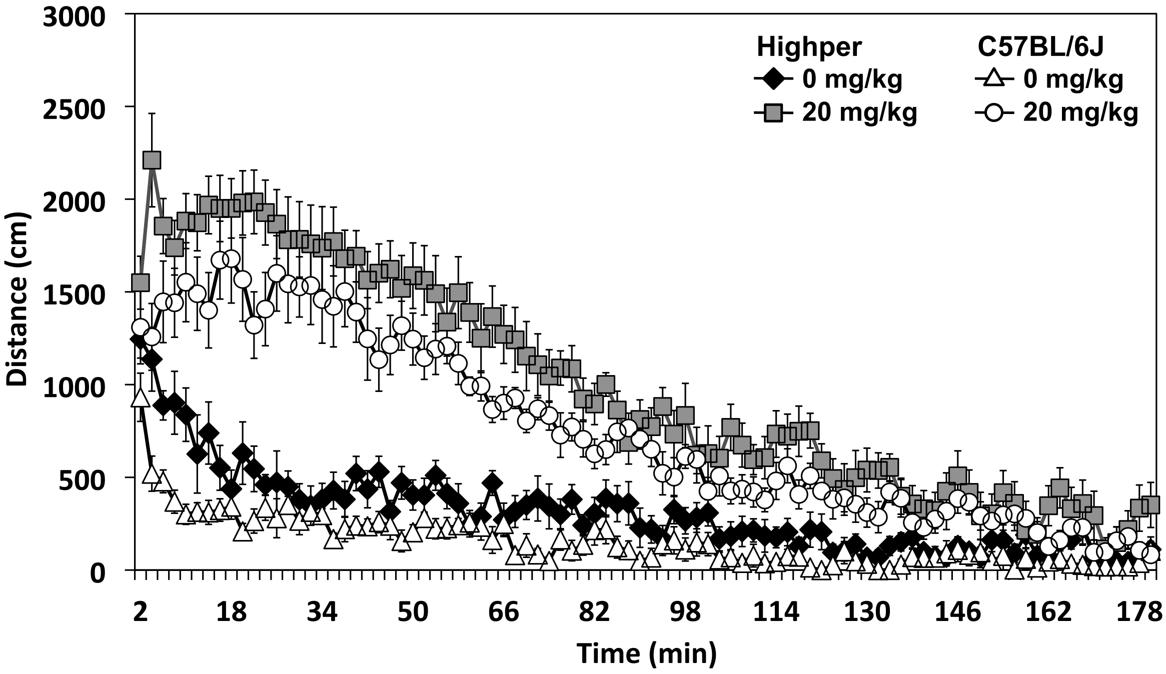

Supplementary Figure 3

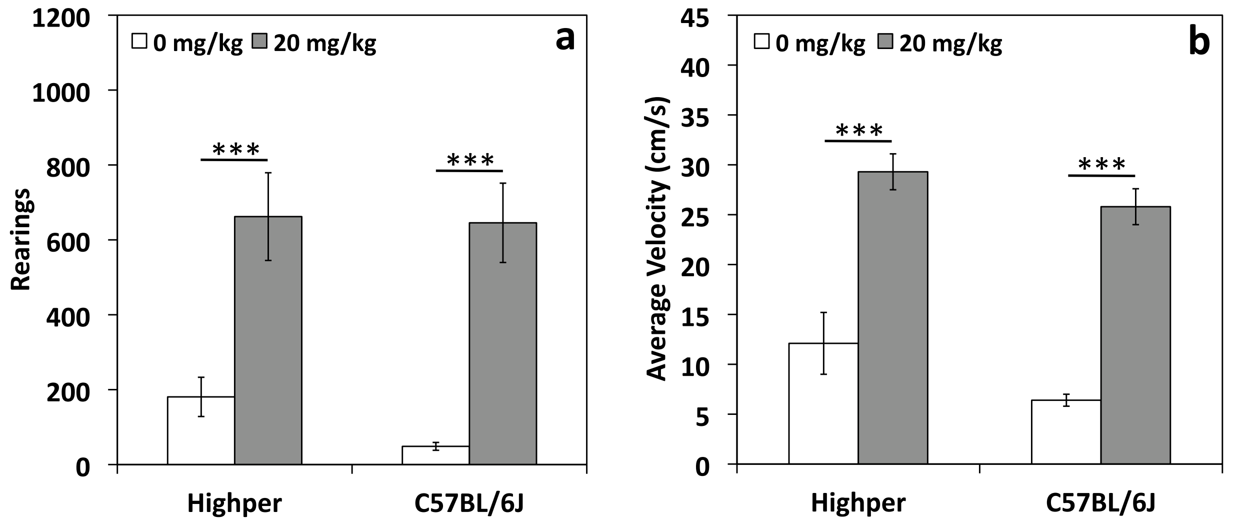

Supplementary Figure 4

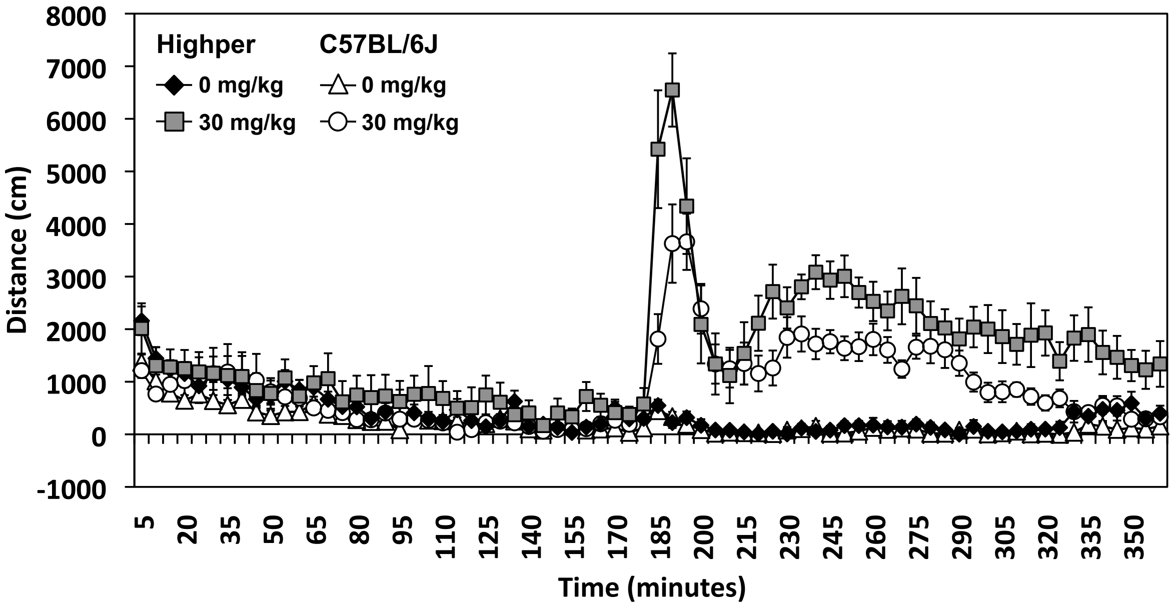

Supplementary Figure 5

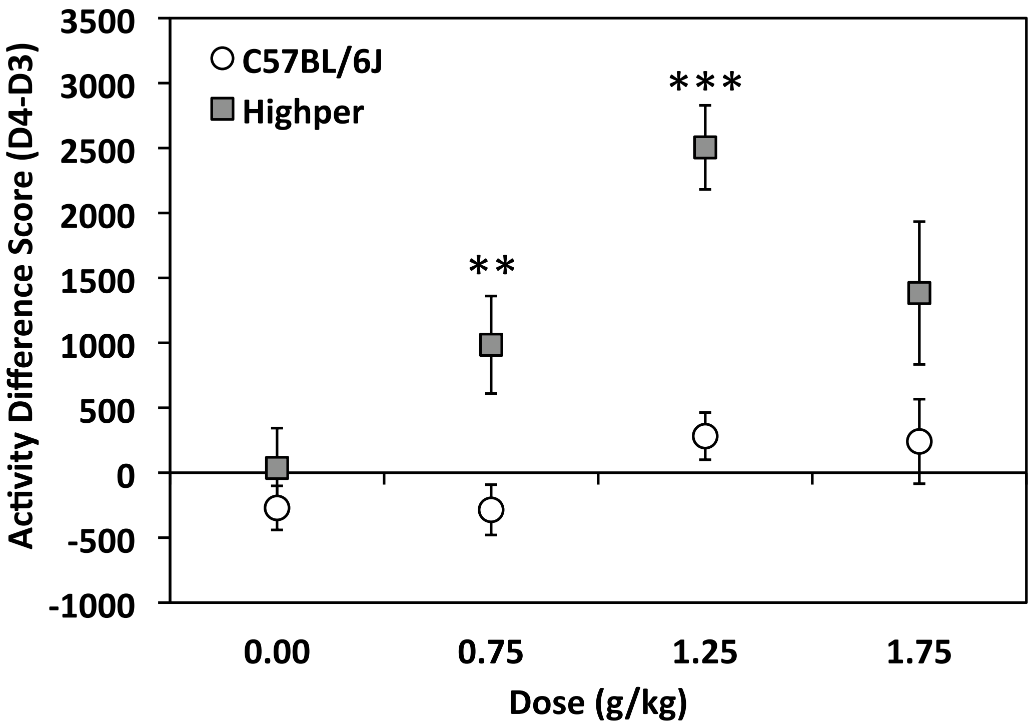

Supplementary Figure 6

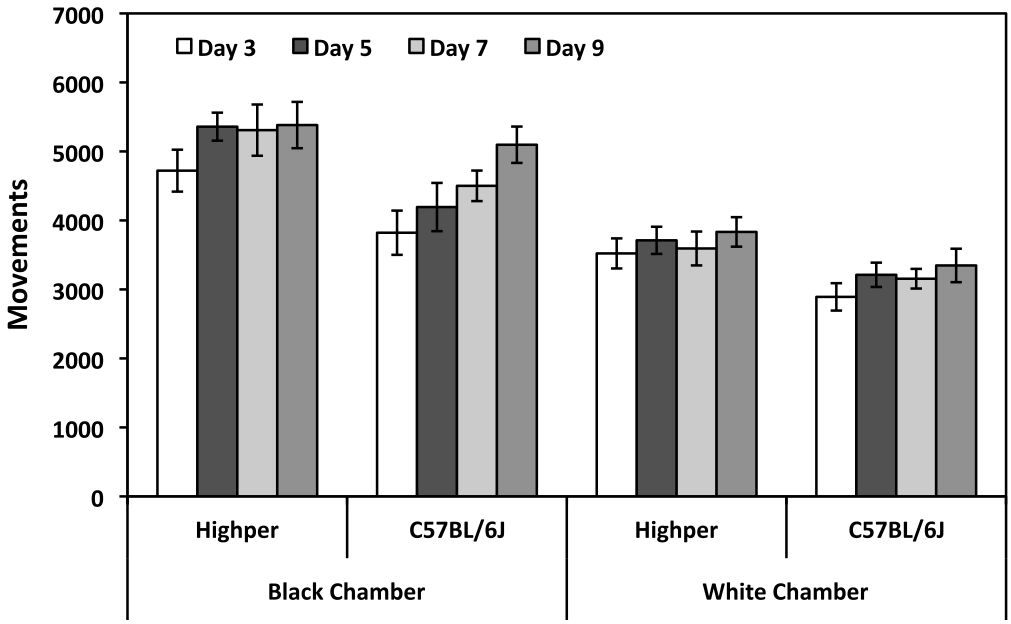

Supplementary Figure 7

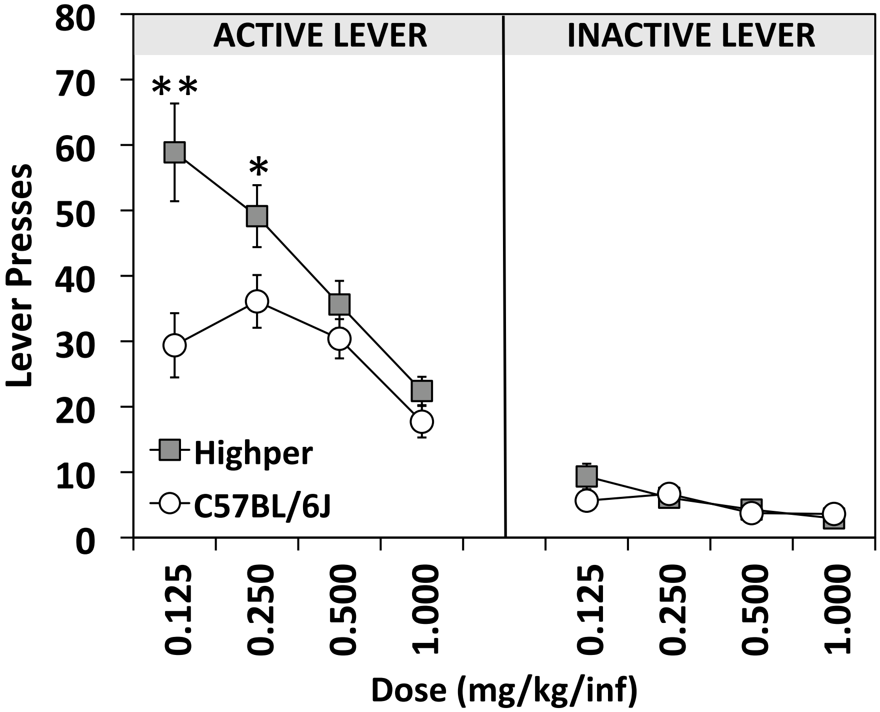

Supplementary Figure 8

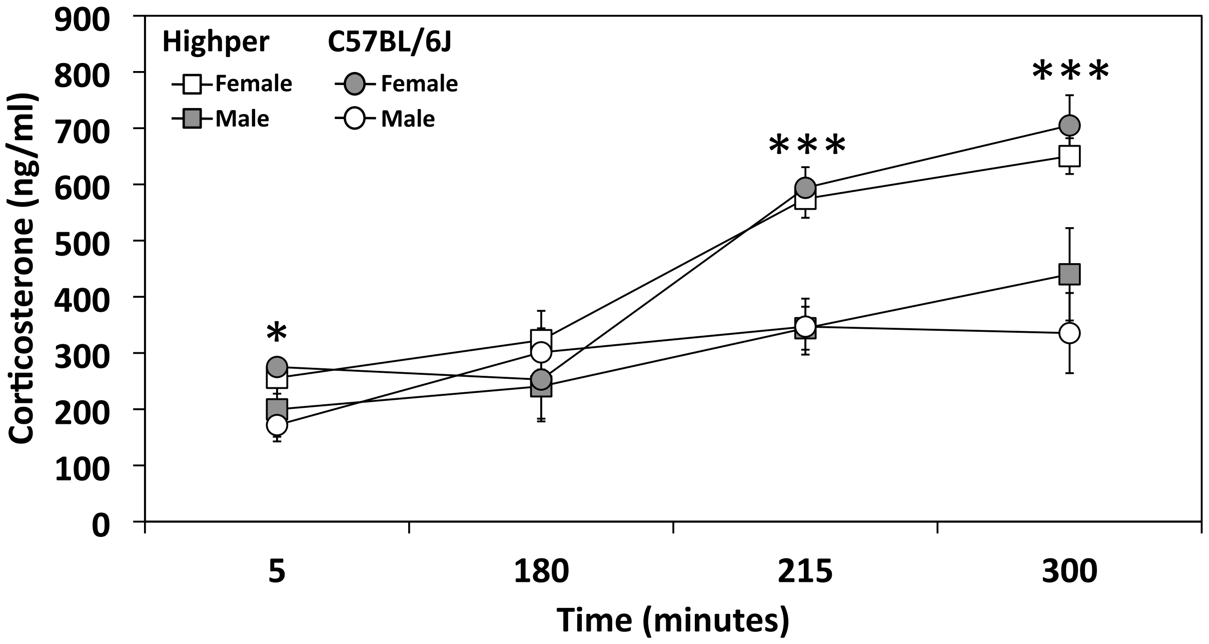

Supplementary Figure 9

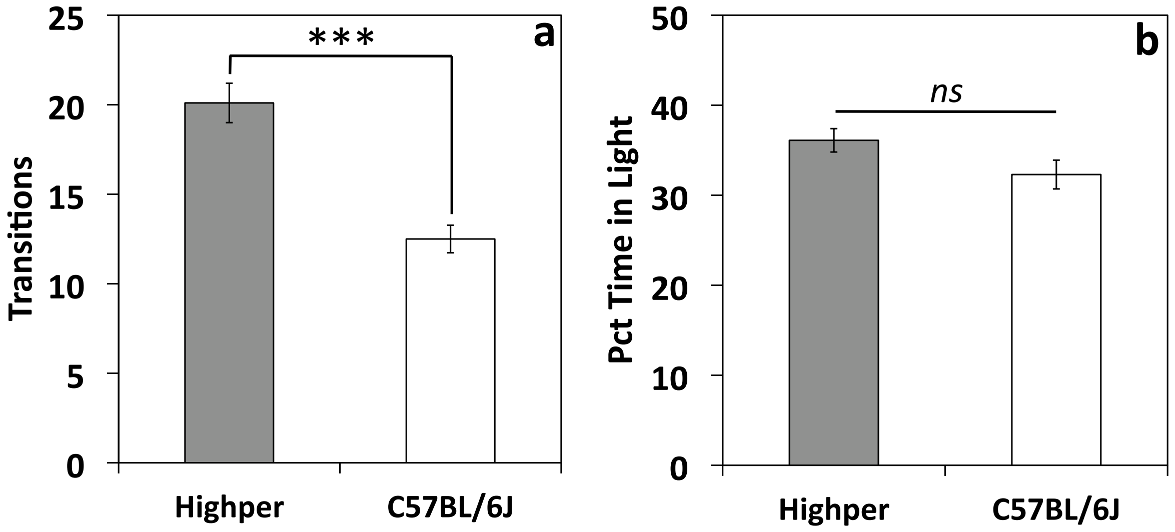

Supplementary Figure 10

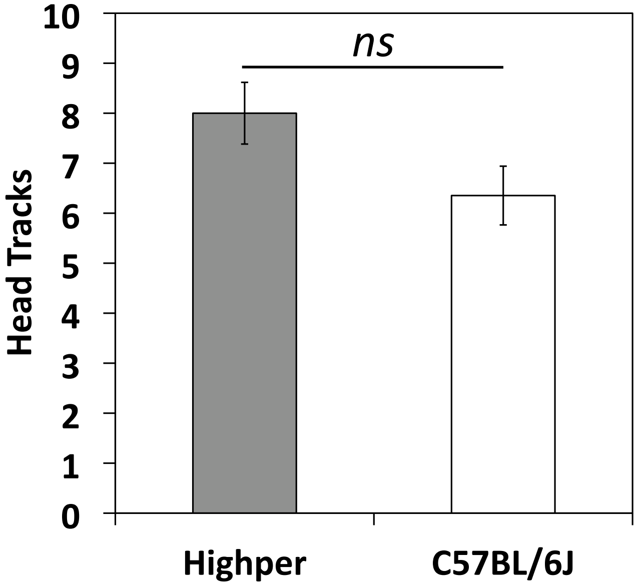

Supplementary Figure 11

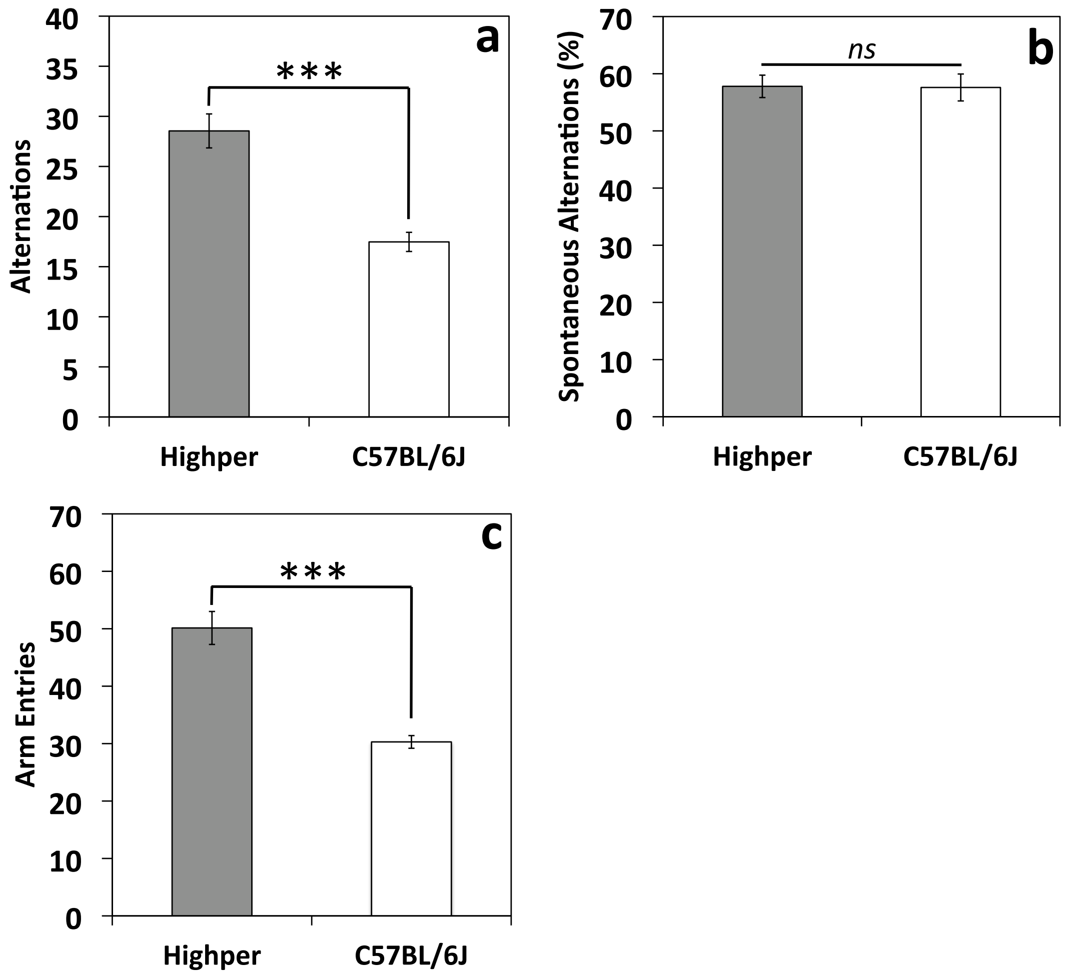

Supplementary Figure 12

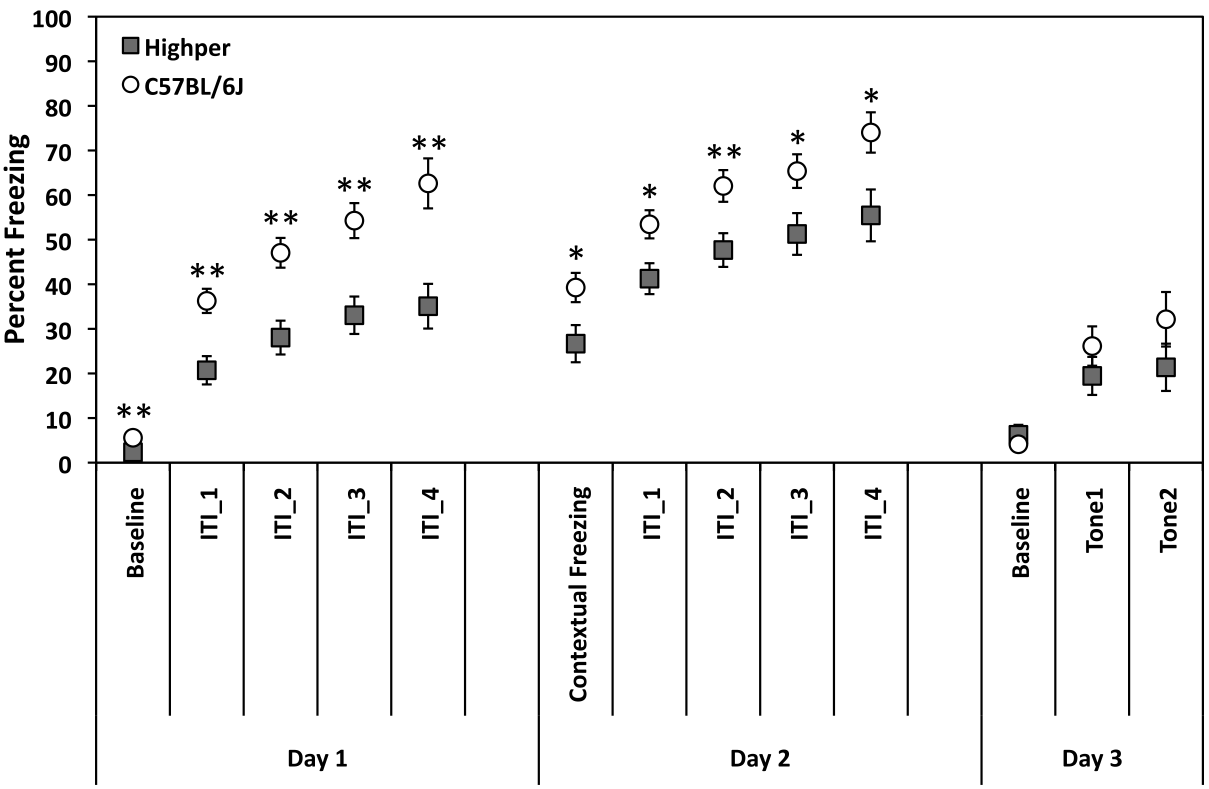

Supplementary References

Crawley J, Goodwin FK (1980) Preliminary report of a simple animal behavior model for the anxiolytic effects of benzodiazepines. Pharmacol Biochem Behav 13: 167-70

Hughes RN (2004) The value of spontaneous alternation behavior (SAB) as a test of retention in pharmacological investigations of memory. Neurosci Biobehav Rev 28: 497-505

Lalonde R (2002) The neurobiological basis of spontaneous alternation. Neurosci Biobehav Rev 26: 91-104

Reijmers LG, Coats JK, Pletcher MT, Wiltshire T, Tarantino LM, Mayford M (2006) A mutant mouse with a highly specific contextual fear-conditioning deficit found in an N-ethyl-N-nitrosourea (ENU) mutagenesis screen. Learn Mem 13: 143-9
